# Patient and Family Involvement: A Discussion of Co-Led Redesign of Healthcare Services

**DOI:** 10.2196/jopm.8957

**Published:** 2018-02-01

**Authors:** Sarah Jane Prior, Steven Campbell

**Affiliations:** ^1^ Rural Clinical School Faculty of Health University of Tasmania Burnie Australia; ^2^ Health Service Innovation Tasmania Faculty of Health University of Tasmania, Hospital Campus Burnie Australia; ^3^ School of Health Sciences Faculty of Health University of Tasmania Newnham Australia

**Keywords:** co-led redesign, health care, patient and family engagement, patient involvement, rural health

## Abstract

The involvement of patients and their families in the redesign of healthcare services is an important option in providing a service that addresses the patients’ needs and improves health outcomes. However, it is a resource-intensive approach, and it is currently not clear when it should be used, and what should be the reasoning behind this decision. Some health systems of international standing have created a patient engagement program as a selling point. This paper discusses how co-led redesign can be beneficial in improving health service and more effectively engaging patients. Potential barriers for patient involvement are discussed. Patient involvement can be integrated into the health system at three main levels of engagement: direct care, organizational design and governance, and policy-making. The aim of this paper is to describe how co-led redesign is compatible with different levels of patient involvement and to address the challenges in delivering a co-led redesign in healthcare. Co-led redesign not only involves the collection of quantitative data for assessing the current systems but also the collection of qualitative data through patient, family, and staff interviews to determine the barriers to patient satisfaction. Co-led redesign is a resource-rich process that requires expertise in data collection and a clinical group that is devoted to implementing recommended changes. Currently, a number of countries have utilized co-led redesign for many different types of healthcare services. Resource availability and cost, process time, and lack of outcome measures are three major limiting factors.

## Introduction

The involvement of patients and their families in the planning and development of health care services has been shown to improve the health and quality of life of patients [1]. Patient and family engagement has been defined as “a relationship between health care providers working together to promote and support active patient and public involvement in health care and to strengthen their influence on health care decisions at an individual and collective level” [2]. Patient and family involvement includes the feedback and experiences from patients and their family members and caregivers. They have experience, expertise, insights and valuable perspectives that are useful in bringing about changes in health care regardless of whether their own experience was positive or negative. Patient engagement, including partnerships, transparency, and information sharing between providers and patients, can be applied to decision making at an organizational level. System modification is required to ensure that patients and families have a voice in planning organisational strategy and in designing changes and improvements in patient care [3].

A number of studies have demonstrated the importance of involving patients and their families in health care redesign initiatives through patient satisfaction surveys or interviews and mapping of patient journeys. Patients in a study of stroke services in the United Kingdom reported high levels of satisfaction with inpatient care [4]. A substantial proportion of patients and their families also reported dissatisfaction with the lack of involvement in decisions [5]. A different patient journey study in the UK [6] showed that four main themes emerged from questioning patients and their families about their in-hospital experiences; (1) information provision, (2) staff attitudes, (3) availability of care, and (4) considering the whole person in context. This paper defines patient involvement in health care and demonstrates the current state of patient and family engagement in the redesign of clinical management for health care services. This paper also highlights the barriers to achieving adequate patient and family engagement in a health care service redesign process.

## Patient Involvement

In 1988, the term patient-centered care was used to call attention to the need for clinical staff and health care systems to shift their focus away from diseases and back to the patient and family [7]. This movement was designed to stress the importance of understanding the patient experience and delivering more effectively on patient needs, including decisions about their care and treatment, diagnostic tests, screening, and medications. This term still refers to a focus on patients, however, it does not necessarily reflect that patients should also be involved in their care or at what level. Patient and family engagement or involvement is now a main driver for improving quality in health care. This engagement can range from consultation to partnership and from a limited decision-making role up to a shared decision-making role. Patient and family engagement has many forms and can occur for a number of reasons. Three levels of engagement for patient involvement, identified by Carman and colleagues [8], are direct care, organizational design and governance, and policy making. This framework is the basis for defining patient and family involvement and co-led redesign in this paper.

## Direct Care

Dieppe and colleagues [9] state that the clinical encounter, the point at which health care professional and patient interact, is “at the heart of health care.” Patient involvement, from a direct care viewpoint, involves including the patient and their family in the decisions that are made about their diagnosis and treatment. It is defined as integrating the patients’ values, experiences, and perspectives in relation to prevention, diagnosis, and treatment [9]. The involvement of patients and their family members in improving the quality of health care has been considered to be a democratic or ethical requirement as patients indirectly pay for services through taxation (in some countries, including Australia) and therefore should have a right to influence how they are managed [10,11]. In the United States, however, employers are often responsible for paying health insurance premiums for their employees’ hospital treatment, often with a copayment from the employee, as per the market-based health insurance system. In this instance, patient involvement might seem more of a priority as there is direct payment for the services utilized. Other ethical considerations, as described by Elwyn and colleagues [12], include individual self-determination and the idea that clinicians need to support this. Self-determination, in the context of shared decision-making and patient and family involvement in health care, pertains to an intrinsic human tendency to preserve one’s own well-being [13], which is something that not all patients or families exhibit. At the direct level, patient and family involvement could mean simply providing patients and their family members with information or involving them actively in setting goals or making decisions about their care.

Information provision was a major theme identified by Morris and colleagues [6] during a patient journey study. Providing patients, and their families and caregivers, with accurate and suitable information is an important component of direct care. Actively involving patients and their families by ensuring they receive and understand information about their condition including treatment has been shown to improve quality of life significantly when compared with patients who did not actively receive this information [14-17]. The Royal Children’s Hospital (Melbourne, Australia) developed a policy that defines patient and family-centered care, including sharing of information, involving the patient and family in decision making, and sharing the provision of care. This direct style of patient involvement is certainly family- and patient-centered but the development of clinical procedures, pathways, and mode of service delivery are still decided by clinicians, with little patient or family input. Currently, many hospitals have policies and procedures that encourage patient and family involvement at the direct level of engagement, but not necessarily in the redesign of the health care services they utilize.

It has been suggested [18] that the most important attribute of patient-centred care is the active engagement of patients when health care decisions must be made. Graffigna and colleagues [19] developed the Patient Health Engagement model which provides an overview of patient engagement. It consists of four stages. Each level addresses a significant stage in the patient journey where the patient becomes a “co-constructer of their health and capable of self-management.” Engaging patients in this way, to allow a sense of control and understanding of their condition, has been shown to have a positive effect on patient satisfaction. It also reduces depression [20]. However, a study by Sommers and colleagues [21] found that some patients would prefer that their health care providers just tell them what to do rather than engage in shared decision making. This suggests that some patients may be less likely to benefit from more collaborative levels of participation. This external aspect of control [22] is a conundrum for proponents of patient involvement, as lower socioeconomic status (SES) patients tend to have less internal control over health. Therefore, while higher SES patients are more likely to take advantage of patient involvement systems, the need is for the lower SES patients to be more involved, and take more control of their health.

## Organizational Design and Governance

Patient involvement in organisational design and governance provides an opportunity for patients to partner with health care providers in planning, delivering and evaluating health care. This encompasses involvement in the design of the health care facility, through to assisting with hiring and training staff. A review of patient and public involvement in health care in the UK was conducted by Mockford and colleagues. They divided the impact of this involvement into service planning and development, information development and dissemination, and changing attitudes of service providers and users [23].

Other forms of patient and family engagement for delivery of improved organizational goals include participation on consumer committees, patient satisfaction surveys, participating in focus groups, and patient and caregiver representation on planning and development boards/panels. Patient involvement at an organizational level also includes participation in quality improvement opportunities. Feedback, including complaints and compliments, can be utilized by management teams to improve the future design of health care services and make changes in governance and policy. Reid Ponte and Peterson [3] suggest that the principles of partnership, transparency, and information sharing must guide the interactions between providers and patients and their families at the bedside, and then be applied to the organizational level.

## Policy Making

Patients, or members of the public, can collaborate with representatives from health care facilities to make decisions about how to shape health care policies and set priorities for the use of resources. Described in the UK as a “remarkable experiment in democratic practice” [24] is a form of patient engagement known as citizen juries. A citizen jury consists of a defined number of carefully selected ordinary citizens who address questions about policy and planning in health care in a primarily advisory role. They are provided information from “witnesses” in their quest to reach consensus around specific health care issues. Although the benefits of a citizen jury include information, time, and independence [25], this process does not provide a real-life account of the experience of the current health care system.

## Co-Led Redesign

Co-led redesign can be defined as “the development and implementation of health care services based on both a clinical and patient perspective and experience or experience-based design.” It involves clinical engagement, patient and family engagement, shared decision making and a thorough analysis of the current systems and expected benefits of new, improved systems. Co-led redesign occurs at all three levels of engagement in differing capacities. In the patient journey clinical redesign process [26], a co-led redesign process, the direct engagement occurs via a systems analysis, performed from the perspective and experience of the patient, and their families. It also includes the front line health care staff who are critical to improving the clinical practice. Organizational design and governance is assessed through a quantitative analysis of data, linked to direct engagement information to form new policy and practice. This evidence-based approach utilizes quantitative and qualitative data to inform the decision makers, at all levels, how to allocate resources and structure health care service provision. Patient involvement in health services redesign is based on the premise that involving patients leads to more accessible and acceptable services and improves the health and quality of life of patients [1]. Mixed method research (combining quantitative and qualitative analysis) can capitalise on the strengths of each approach. This includes corroborating findings, generating more complete data, and using the results from one method to enhance the insights from the other. In the UK, government policy states that “involving patients and the public isn’t always easy and can take time but, done well, has been shown to be highly effective in developing services that better meet patient needs and lead to better health outcomes” [27]. There is evidence to suggest that patient engagement in the redesign of health care services is linked to fewer adverse events, better patient self-management, fewer diagnostic tests, decreased use of health care services, and shorter lengths of stay in hospitals [28]. Experience-based design is a user-focused design process with the goal of making user experience accessible to allow design of a better patient and staff experience [29]. Co-led redesign places the experience goals of patients and their families and staff at the centre of the design process. It creates a partnership with the patients, families and staff, and promotes shared leadership and decision making.

## Use of Patient Engagement within Redesign and Co-Led Redesign Models

Instances of direct engagement, organisational design and governance changes and policy making can be shown through varying types of patient involvement initiatives locally and globally. A national survey of hospitals in the United States [28] reports that of the 1457 hospitals that responded, 7% include patients and family members in the education and content development when training clinical staff, 21% had a patient and family advisory council that had met within the previous 12 months and 23% had patient and family advisory councils [28]. This type of patient and/or family involvement is not necessarily for health care redesign purposes, although it does provide an opportunity for patients and families to give input regarding various hospital activities. This suggests that while patient and family engagement is occurring, the level of participation is inadequate for a patient journey co-led redesign process, which requires more in depth, personal patient and family involvement. Although this information is valuable and the economic impact of utilizing patients and their families in the redesign of health care services has been shown to be a limiting factor, particularly when interviewing individual patients and families is chosen research method [23].

Co-led redesign incorporates the patient and family feedback and suggestions on how to improve current services based on previous experience in a particular health service in conjunction with other research methods. The qualitative method of interviewing patients and their families allows them to identify gaps and strengths of the system and influence the redesign of health care services. Patient and family feedback is utilized to directly influence changes to be made to existing services or to the development of new services. A systematic review of involving patients in planning and development of health care services [30] reported that patients who participated in these initiatives welcomed the opportunity and that their self-esteem improved as a result of their contribution. This review suggests the most frequently reported effects of involving patients in developing or improving health services include making services more accessible through simplifying appointment procedures, extending opening hours, improving transport to treatment units, and improving access for people with disabilities. Patient involvement may take place via face-to-face meetings, patient representation in planning meetings, group interviews, written surveys and consumer boards [30].

Collection of qualitative data in co-led redesign, through direct engagement methods, patient interviews, or other interactive forums, is time consuming and resource rich and its subsequent analysis is much the same. Qualitative methods generate a substantial amount of data, it is suggested that just 20 one-hour interviews can generate up to 400 single spaced pages of transcripts [31]. Thematic analysis has been shown to be effective in identifying gaps in service, areas for overall improvement and barriers to effective service delivery despite the timely analysis process [32]. Identifying themes and patterns from the experiences of patients and their families ensures a comprehensive view of the overall service but involves a number of time consuming components. Themes are defined as units derived from patterns such as conversation topics, vocabulary, recurring activities, meanings, feelings, or folk sayings and proverbs [33] and are often determined from recorded interview transcripts. These can then be divided further into sub-themes for identifying further patterns in data. Following theme definition, a literature review should be performed to validate the argument for the choice of themes (and sub-themes) in order to build a report, or story that highlights the patient perspective on the health service. Broad outcome measures for quantitative analysis, such as length of stay and readmission rates, are useful across many conditions. However, developing condition-specific measures, (effective interventions to improve the quality of care for qualitative analysis in co-led redesign becomes a time consuming and difficult process. Both methods of research have benefits in co-led redesign. Quantitative research counts occurrences (eg, prevalence, frequency), whereas qualitative research, following a thorough analysis, can describe the complexity, range of or breadth of occurrences and generate hypotheses about a particular phenomenon [31].

New South Wales Health (Australia) and Flinders Medical Centre (South Australia) underwent a major clinical services redesign program between 2002 and 2005 [34] utilizing the patient journey method. Mapping of the current patient journey was performed by involving all staff members as well as interviewing patients and their families about their experiences within particular health services. This provided an avenue for patients and their families to reflect on the service, provide feedback about potential improvements, and gave them an opportunity to speak openly about strengths and weaknesses of the overall health care service. The clinical services redesign in New South Wales is ongoing and is delivered as part of the NSW Agency for Clinical Innovation Program [35]. The benefits of the Flinders Medical Centre redesign include the stabilizing of staffing, reductions in the numbers of adverse events throughout the hospital and reduced length of stay for medical patients admitted as emergency cases [34]. In the United Kingdom, the involvement of patients and the public in shaping health care is well established in National Health Service (NHS) policy and reinforced by a government that is committed to empowering individuals to play a greater role in their own health care [36]. The type of redesign developed within the NHS has been utilized for a number of health care services including prostate cancer, acute coronary syndrome, cholecystectomy and head and neck cancer. The prostate cancer redesign resulted in changes that could not have occurred without using a co-design process with [patient] interviewees, such as through the support group that was developed. Data from the prostate cancer redesign indicated that appointments were not coordinated for the patients and their family. A solution that was proposed was a one-stop-shop for all diagnostic tests, but the men and their wives [from the support group/ from the interviews] considered that all of the tests being done on the one day would be too much. [26].

Co-led redesign has also been successfully implemented in services in Sweden, where patients with diabetes were consulted formally about the existing diabetes management in primary care plans. This led to changes to the organization of care and in the type of information provided to patients utilizing this service [37]. Between 2004 and 2008, a patient-centered method to redesign patient care delivery was developed and refined at the University of Pittsburgh Medical Center (US) as a means of improving patient care experiences and exceeding the needs and desires of patients and their families [38]. This was a co-led redesign process involving the selection of a particular patient experience (health service), establishment of a patient and family-centered care experience group, the mapping of the complete patient journey (surveys, storytelling, patient shadowing, and family experience), and the involvement of all staff in the care experience. This initiative resulted in a dramatically improved service and outcomes without increasing cost. It also eradicated silos that are often seen in hospital systems. Results include a 14% significant increase in patient satisfaction in the emergency department and a 13% significant increase in patient satisfaction in the general trauma ward [38]. The savings in this study can be attributed to the development of patient and family experience initiatives based on the timeline of implementation and evaluation. This study also resulted in a decrease in staff turnover of 66% over three years, as well as an annual saving of $5,000 in one inpatient unit by changing the late food-tray menu and process.

## Barriers to the Use of Co-Led Redesign

### General Barriers

While co-led redesign has many benefits, it also has some limitations. The staff resources required to complete the quantitative and qualitative analysis adequately on behalf of the health service can create delays and can be quite costly, resource-wise and financially. Completion of this work by health care staff may also create staff stress and anxiety if they feel inexperienced and lack time and resources. Co-led redesign involves the establishment of a patient journey group to oversee the process and take on the responsibility of ensuring that the redesign project is understood and working well. The patient journey group comprises a number of roles including a chair, lead, and a clinical champion, all coming from within the organization and a patient journey facilitator who is not a part of the clinical team [26]. Knowledge management is a key part of the co-led redesign process. Regular meetings and communication of research findings, progress reviews, maintaining focus, and delivering on the expectations of the redesign are important in ensuring overall success. Again, time and resources often don’t allow key players to be fully invested in co-led redesign, despite their intentions for change. While a number of patient and family engagement initiatives have been developed in many health care organizations outside of co-led redesign, they often lack clear guidelines and fail to reveal an evidence-base to explain or support the approach [39].

Some potential barriers for overall consumer and patient involvement in health care and general redesign initiatives were identified by Nilsen and colleagues [40]. Health professionals often view themselves as authorities. People may believe that involving patients in policy, research, and practice increases costs and causes delays. They may also fear that patients may have biased views that interfere with the “academic impartiality” of knowledge development [41]. Another factor found to impede meaningful patient involvement is organizational and professional resistance to change or learning something from health service providers [42]. Evidence suggests that a blame culture within health care organizations prevents staff from being open and sharing their views [43-46]. Managerial interest is often focused on budgets and targets and achieving status rather than on patients and their families. There is also evidence to suggest that staff shortages, lack of time and resources, poor communication, and fragmented ways of working continue to affect both patient and staff experiences adversely [43,46-48]. Issues based on the patient and staff experience with care, and the delivery of care in general, are important for understanding potential issues within co-led redesign. It is imperative to ensure that the patient and family experience and story is not demeaned by a lack of managerial support for co-led redesign, or that a focus on administrative targets prevents the full involvement of patients and their families.

### Health Literacy

Another major limiting factor in direct engagement of patients and their families is low health literacy, particularly in areas known to have a low SES. Initial and ongoing patient participation in the qualitative component of co-led redesign is dependent on a number of patient-specific issues, as identified by Jordan and colleagues [49]: the ability to identify and understand health messages, having access to information and services, and possessing the skills to decide which information is useful. A key limiting factor for active patient participation in developing and building relationships with health care providers is health literacy of the patient and their family. Education and health literacy potentially limit a person’s ability to be involved in decisions about their health [50] and the health care of their families. Greater involvement places an increased demand on each patient’s literacy skills in order to understand complex health information and articulate their preferences and their experiences [50]. Co-led redesign relies on information directly from the patient and their families through directly asking the patient about their experience. Health literacy levels have been shown to influence this information. In a study by Smith and colleagues [50] patients with a lower health literacy level reported that they were not interested in trying to understand the “mechanics or you know, pros and cons”. These patients were more interested in having their doctor take the lead and offer a definitive decision. Patients with a higher health literacy, reported seeking independent knowledge around their condition, although still respected their doctor’s expertise. While not directly related to health literacy it is also important to consider and establish a method of meaningful communication with patients and family members who may have communication impairments such as aphasia, deafness or some forms of mental illness. To overcome this issue, it is important to recognize the differing health literacy levels in individuals and present the information accordingly. In order to empower patients and their families who have low health literacy levels, and give them the opportunity to participate in co-led redesign, all contact should be made personally and in a manner that creates an environment where questions are welcome and information can be understood.

Poor health literacy and low SES tend to go hand in hand, as does chronic disease and low SES [51][52]. Gaining informed consent from this group in co-design is a challenge, but they are the very people who need to take part. Informal approaches to such potential participants, such as phone calls, can open up fruitful participation, as opposed to a formal letter. Focus groups may be a good way to gain views from higher SES patients with good health literacy, but can be very threatening for other groups. The venue for an interview may provide a solution by changing the power relationship. For example, offering to do the interview in the patient’s home, with supportive family members or friends present, may convince him to consent. The style of the interview is important in terms of using the appropriate level of English and being clear about the concepts concerned. An interviewer who creates an environment in which the participant knows that there is no right or wrong answer, and that his or her views are valued, is crucial.

### Complaints Versus Feedback

Another barrier to successful patient led initiatives around improving health care services, identified by Mead and Bower [53] is the tension between the aims and priorities of health practitioners and those of the patients and families. This conflict has been identified as a limitation in the organisational design and governance engagement level as well as from a policy making perspective. If there is a specific issue or complaint from a patient or family member, this may be the only focus of their involvement and they may become distracted from the overall feedback process. The involvement of patients and families with their own agendas for taking part in the research project may be counterproductive as their attention is on one aspect of service and they may fail to become engaged in the overall feedback journey. An interviewer can ensure that the direction of the conversation remains focused on experience feedback and relevant information by using a semi-structured questioning technique, allowing the interviewer to bring the conversation back when required.

## Conclusion: Future of Co-Led Redesign

Co-led redesign has been shown to have a number of benefits over traditional health care redesign initiatives, most notably, adding the patients’ (and their family’s) perspective to particular health care services. This information not only provides another source of evidence to build a case for redesign, but it also ensures that patients and their families are able to share their unique experiences, are represented, and feel involved in the improvement of the delivery of their health care services. There are many successful redesigned services around the world that are a result of a co-led approach. This suggests that this method may be a major option for the future of clinical redesign.

But, without serious investment in an infrastructure for co-led redesign, as well as a commitment from its leadership and management, it is not possible for most health systems to adopt co-led redesign as their standard approach. Indeed, even a local single service co-led redesign is resource intensive, requiring a clinical team that is open to being informed in this way. It can be a challenge to find staff or university partners with the skills to bring in the patient voice. Clearly, there is much more work that needs to be done to fully develop a co-led redesign model for health care.
